# Maternal gut microbiota during pregnancy and the composition of immune cells in infancy

**DOI:** 10.3389/fimmu.2022.986340

**Published:** 2022-09-21

**Authors:** Yuan Gao, Martin O’Hely, Thomas P. Quinn, Anne-Louise Ponsonby, Leonard C. Harrison, Hanne Frøkiær, Mimi L. K. Tang, Susanne Brix, Karsten Kristiansen, Dave Burgner, Richard Saffery, Sarath Ranganathan, Fiona Collier, Peter Vuillermin

**Affiliations:** ^1^ School of Medicine, Deakin University, Geelong, VIC, Australia; ^2^ Child Health Research Unit, Barwon Health, Geelong, VIC, Australia; ^3^ Faculty of Science, Copenhagen University, København, Denmark; ^4^ Murdoch Children’s Research Institute, Royal Children’s Hospital, Melbourne, VIC, Australia; ^5^ Independent Scientist, Geelong, VIC, Australia; ^6^ The Early Brain Science Department, Florey Institute of Neuroscience and Mental Health, Melbourne, VIC, Australia; ^7^ Population Health and Immunity Division, Walter and Eliza Hall Institute of Medical Research, Melbourne, VIC, Australia; ^8^ Department of Medical Biology, University of Melbourne, Melbourne, VIC, Australia; ^9^ Department of Pediatrics, University of Melbourne, Melbourne, VIC, Australia; ^10^ Department of Biotechnology and Biomedicine, Technical University of Denmark, Kongens Lyngby, Denmark; ^11^ Laboratory of Genomics and Molecular Biomedicine, Department of Biology, University of Copenhagen, Copenhagen, Denmark

**Keywords:** birth cohort, gut microbiota, maternal microbiota, fetal immunity, neonatal T cells

## Abstract

**Background:**

Preclinical studies have shown that maternal gut microbiota during pregnancy play a key role in prenatal immune development but the relevance of these findings to humans is unknown. The aim of this prebirth cohort study was to investigate the association between the maternal gut microbiota in pregnancy and the composition of the infant’s cord and peripheral blood immune cells over the first year of life.

**Methods:**

The Barwon Infant Study cohort (*n*=1074 infants) was recruited using an unselected sampling frame. Maternal fecal samples were collected at 36 weeks of pregnancy and flow cytometry was conducted on cord/peripheral blood collected at birth, 6 and 12 months of age. Among a randomly selected sub-cohort with available samples (*n*=293), maternal gut microbiota was characterized by sequencing the 16S rRNA V4 region. Operational taxonomic units (OTUs) were clustered based on their abundance. Associations between maternal fecal microbiota clusters and infant granulocyte, monocyte and lymphocyte subsets were explored using compositional data analysis. Partial least squares (PLS) and regression models were used to investigate the relationships/associations between environmental, maternal and infant factors, and OTU clusters.

**Results:**

We identified six clusters of co-occurring OTUs. The first two components in the PLS regression explained 39% and 33% of the covariance between the maternal prenatal OTU clusters and immune cell populations in offspring at birth. A cluster in which *Dialister, Escherichia*, and *Ruminococcus* were predominant was associated with a lower proportion of granulocytes (*p*=0.002), and higher proportions of both central naïve CD4^+^ T cells (CD4^+^/CD45RA^+^/CD31^−^) (*p*<0.001) and naïve regulatory T cells (Treg) (CD4^+^/CD45RA^+^/FoxP3^low^) (*p*=0.02) in cord blood. The association with central naïve CD4^+^ T cells persisted to 12 months of age.

**Conclusion:**

This birth cohort study provides evidence consistent with past preclinical models that the maternal gut microbiota during pregnancy plays a role in shaping the composition of innate and adaptive elements of the infant’s immune system following birth.

## Introduction

Early life is a critical window during which a range of modifiable factors influence immune development and, in turn, the risk of infectious, allergic and autoimmune diseases. Animal studies have shown that maternal gut microbiota play a non-redundant role in the development of the fetal innate and adaptive immune system. For instance, transient colonization of germ-free pregnant mice with a *Escherichia coli* strain profoundly increased intestinal innate lymphoid cells (NKp46+RORgt+ ILC3 subset) and mononuclear cells (F4/80+CD11c+) in pups, and reduced susceptibility to infection (1). Metabolic products derived from the gut microbiota during gestation have also been shown to impact the development of fetal adaptive immunity. Specifically, offspring of germ-free mice are born with a marked deficit in thymus derived CD4^+^ T cells, including regulatory T cells (Treg), which can be rescued by supplementing the maternal drinking water with acetate, a key microbial metabolic product (2).

Changes in the composition and metabolic activity of the maternal gut microbiome during pregnancy, and their impacts on innate and adaptive immune ontogeny, may be one factor driving the increase in immune-related diseases among children in the modern environment (3). Indirect evidence from epidemiological studies indicates that the maternal gut microbiome is likely to impact fetal immune development. For example, maternal exposure to farm animals and intake of unpasteurized milk during pregnancy are each associated with greater numbers and functionality of Treg in cord blood, increased *FOXP3* locus demethylation, lower Th2 cytokine secretion and lymphocyte proliferation (4), and higher expression of innate immune receptors (5). Similarly, exposure to farm animals during gestation is associated with a higher proportion of Treg in cord blood (6). Although it is considered likely that these associations are partially mediated by the composition and metabolic products of the maternal gut microbiome (3, 7), there are currently no published human studies directly relating the composition of maternal gut microbiota during pregnancy to the infant’s immune phenotype.

We identified clusters of co-occurring operational taxonomic units (OTUs) in fecal samples collected from women during the third trimester of pregnancy, and then investigated associations between these OTU clusters and the composition of the infant’s cord and peripheral blood measured by flow cytometry. Our findings indicate that maternal gut microbiota likely impact the proportion of innate and adaptive cells at birth, including key regulatory populations.

## Methods

### Study population

**Figure 1:**
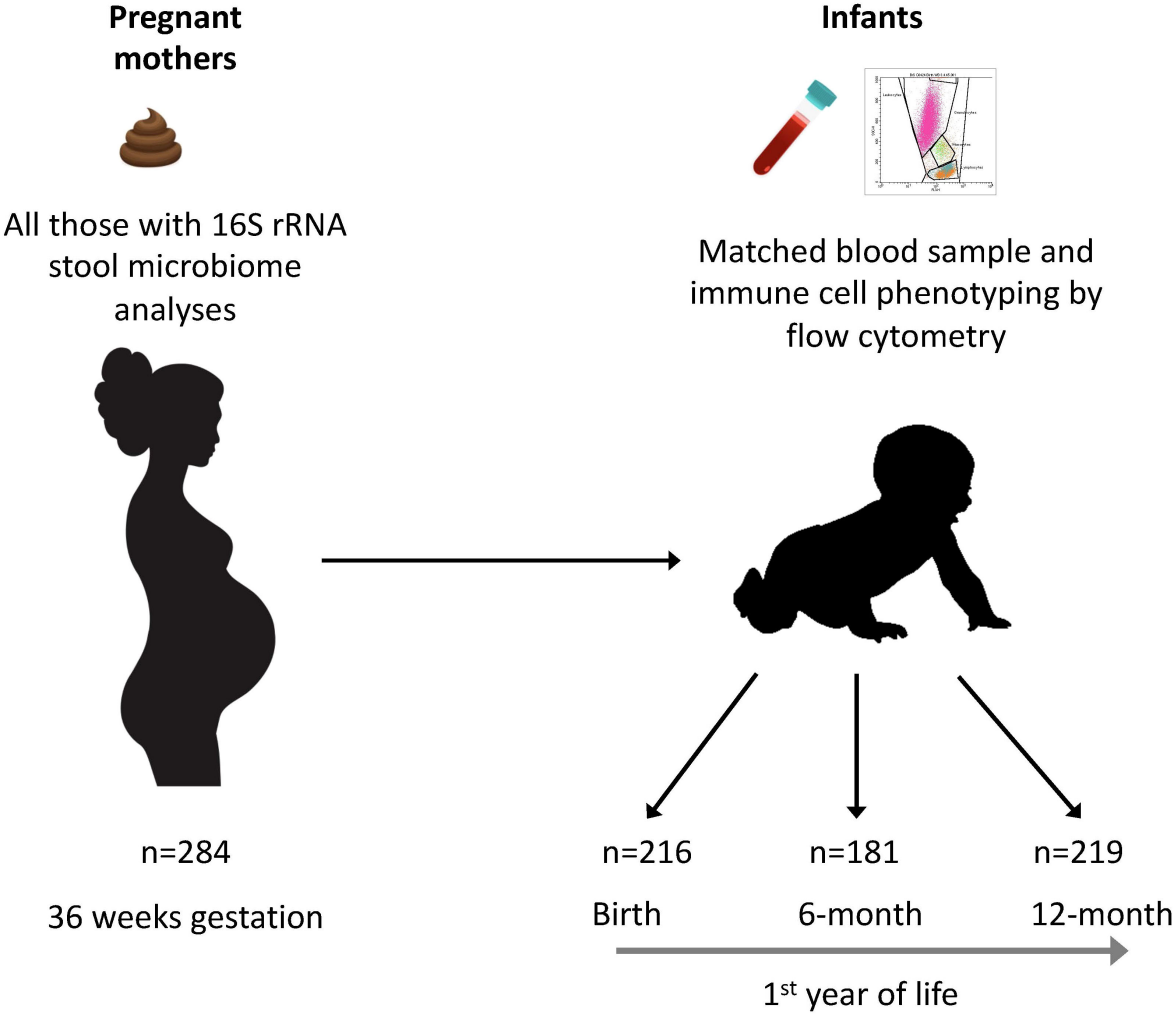
Participants and samples included in the study. Fecal samples from 284 mothers were collected at 36 weeks of gestation and the microbiome was analyzed by 16S rRNA gene amplicon sequencing. Cord blood samples from 216 neonates at birth, and peripheral blood samples from 181 infants at 6 months of age and 219 infants at 12 months were analyzed by flow cytometry.

The Barwon Infant Study (BIS) is an Australian birth cohort study (inception cohort: *n*=1064 mothers/1074 infants) recruited using an unselected antenatal sampling frame (8). Fecal samples were collected from pregnant women at 36 weeks gestation. Cord blood was collected at birth, and peripheral blood samples were collected from infants at 6 and 12 months. Maternal age, body mass index (BMI), antibiotic exposure, parity, household size, and ownership of pets or livestock during pregnancy were recorded by questionnaire at enrolment (28–32 weeks gestation) (**Table S1**). Gestational age at birth, infant sex, mode of birth, birth weight, and feeding practice were recorded at, or after, birth. A random subcohort of 321 families was selected for unbiased investigations; the present study is based on 286 mother-infant pairs (including 2 pairs of twins) for whom both maternal fecal 16S sequence data and infant immune measures from at least one of birth, 6 and 12 months were available (**Figures 1**, **S1**). The study was approved by the Barwon Health Human Research and Ethics Committee (HREC 10/24).

### Fecal collection and processing

Fecal samples from 36 weeks gestation were delivered to the laboratory either frozen (−20°C home freezer) or fresh (if collected within 4 hours) and after aliquoting they were stored at −80°C until analysis. DNA was extracted using the Qiagen PowerSoil^®^ DNA Isolation Kit, Cat#12888-100 and transported on dry ice to the J. Craig Venter Institute, Rockville, MD, USA. Universal primers for the V4 region of the 16S rRNA gene were used to amplify a 292 bp product that was sequenced on the Illumina MiSeq platform. USEARCH (9) software was used to cluster paired-end reads into OTUs. The mothur (10) software suite was used to assign representative sequences to taxa in the SILVA v123 Nr99 taxonomic database, as described in Vuillermin et al. (11).

### Blood collection and processing

Umbilical cord blood (30 mL) was collected by syringe and immediately added to 20 mL RPMI 1640 (Gibco, Life Technologies) containing preservative-free sodium heparin (Pfizer, 10 IU/mL). Venous peripheral blood was collected at 6 and 12 months of age and added to a 15mL tube with preservative-free sodium heparin (10 IU/mL). Blood samples were maintained at room temperature on a roller until processed (within 18h of collection). Mononuclear cells (MNC) were isolated by density gradient centrifugation (Lymphoprep, AxisShield) and immune populations in both whole blood and MNC were analyzed by flow cytometry.

#### Flow cytometry of immune cells

Immune cell populations were analyzed by flow cytometry (**Table S2**) using a 3-channel flow cytometer (FACSCalibur, Becton Dickinson). Antibodies were purchased from BD Biosciences (San Jose, California). Isotype controls were used to set up the instrument and the positive gating with these settings was maintained throughout. Initially samples of whole blood were stained with anti-human CD3-FITC, anti-human CD4-PE and anti-human CD45-PerCP before lysis of the red blood cells (BD FACS Lysing Solution), PBS wash, and fixation in 2% formalin. The proportions of granulocytes, lymphocytes and monocytes as a percentage of total white blood cells (WBC) were discriminated based on side scatter (SSC) and CD45 expression (**Figure S2A**). The proportion of CD3^+^ T cells, CD4^+^ T cells and CD8^+^ T cells were then assessed by gating on the lymphocyte population (**Figure S2B**).

To discriminate thymic and central naïve CD4^+^ T cells subsets, a sample of the isolated MNC was then stained as previously described (12) with anti-human CD4-FITC, anti-human CD31-PE and anti-human CD45RA-PECy5. Cells were gated to the CD4^+^ lymphocytes and divided into flow plot quadrant one (q1), CD31^−^/CD45RA^+^ (central naïve); quadrant two (q2), CD31^+^/CD45RA^+^ (thymic naïve); and quadrant three (q3), CD31^−^/CD45RA^-^ (memory) (**Figure S2C** and **Table S2**) (13).

To discriminate Treg subsets, a sample of the isolated MNC was stained as previously described (12) with anti-human CD4-PE and anti-human CD45RA-PECy5. After the primary antibody staining, samples were washed in PBS and fixed in 2% formalin. After overnight fixation, cells were permeabilized (0.5% Tween) and stained with anti-human FoxP3-Alexa Fluor^®^ 488 (**Figure S2B**). Cells were gated to the CD4^+^ T cells and categorized according to CD4^+^/CD45RA^+^/FoxP3^low^ naïve Treg (nTreg), and CD4^+^/CD45RA^−^/FoxP3^high^ activated Treg (aTreg), non-suppressive FoxP3^+^ T cells (CD4^+^/CD45RA^−^/FoxP3^low^) and FoxP3^neg^ T cells (CD4^+^/FoxP3^neg^) (**Figure S2D** and **Table S2**) (13).

### Short chain fatty acid (SCFA) measurement

Maternal serum samples collected at 28 weeks of gestation and fecal samples collected at 36 weeks of gestation (as described above) were transported at −80°C to the CSIRO (Commonwealth Scientific and Industrial Research Organization) laboratories, Adelaide, Australia. Three SCFAs, acetate, propionate and butyrate, were quantitated using capillary gas chromatography (GC; 5890 series II Hewlett Packard, Australia). Researchers were blinded to the experimental group.

### Statistical analysis

We reduced the dimensionality of the microbiome data using prevalence filtering and *k*-means clustering, resulting in six clusters of co-occurring OTUs. The OTU counts were added together [called amalgamation c.f (14)], then CLR-transformed. CLR-transformation was shown to be essential in microbiome analyses (15, 16). These clusters represent co-occurring populations of bacteria and are distinct from, and should not be confused with, gut microbiota enterotypes, for reasons discussed in detail in **Supplementary Methods**. Next, we pre-processed the immune cell populations, which were considered to be compositional data (**Supplementary Methods** and **Table S2**). We standardized the data using the centered log ratio (CLR) transformation, which formed the immune profile dataset.

**Figure 2:**
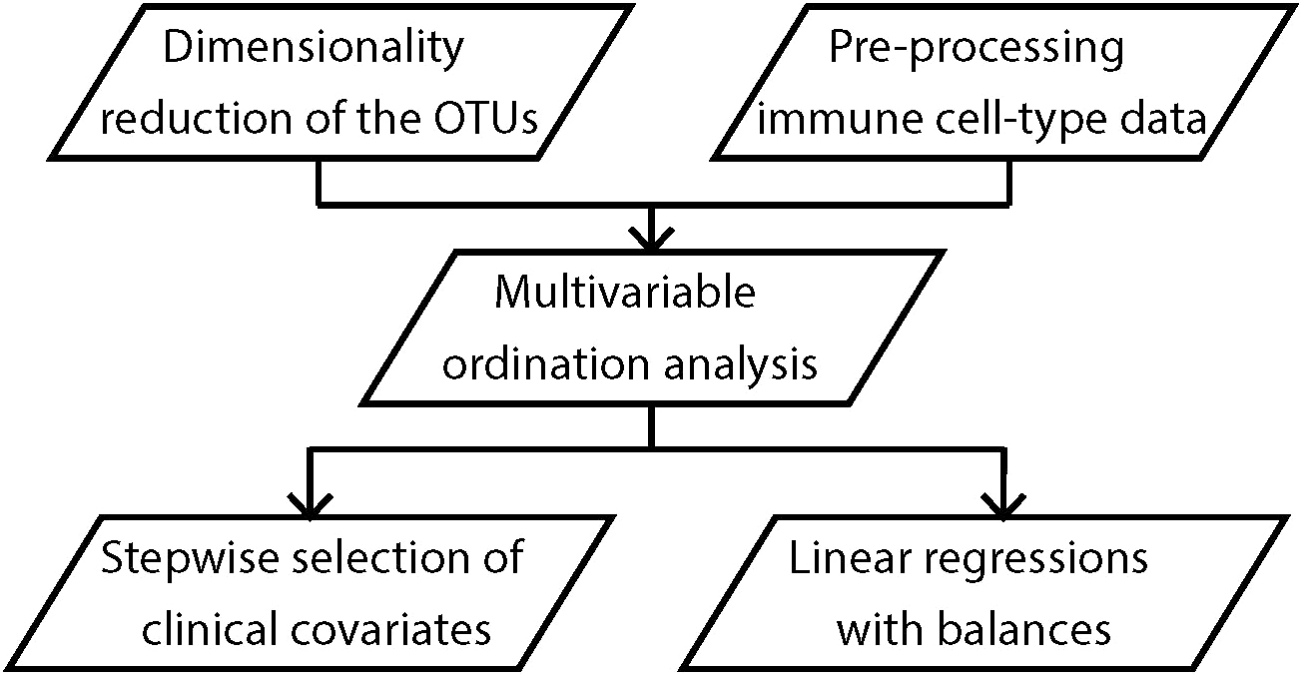
Analysis workflow. All analyses were performed on maternal OTU clusters and/or infant immune profile at birth, 6 and 12 months of age.

Analyses (**Figure 2**) were performed initially for the subsets of granulocytes (gos), monocytes (mos), CD3 negative lymphocytes (CD3^-^), CD8^+^ T cells (CD8), and CD4^+^ T cells (CD4). We explored the associations between maternal OTU clusters and infant immune phenotypes at birth using partial least squares (PLS) regression. We then applied forward stepwise selection of clinical covariates on the first two PLS components to identify the covariates that explain the shared variation between the maternal and infant immune datasets. Finally, we fitted multi-dimensional linear regression models against the clinical covariates and immune profile on OTU clusters, adjusting for potential confounders. Potential confounding factors, including sex, household size, maternal age, maternal BMI, pet or livestock ownership, and use of antibiotics during the third trimester were selected based on the disjunctive cause criterion (17) and those that changed estimates by more than 10% were included in the regressions. The slopes (*β*), 95% confidence intervals (CI) and p values are provided. Secondary analyses including PLS regression and multi-dimensional linear regression models were performed on naïve subsets of the CD4 including central naïve CD4^+^ T cells (q1), thymic naïve CD4^+^ T cells (q2) and CD4^+^ memory T-helper cells (q3), and Treg subsets including FoxP3 naïve Treg (nTreg), FoxP3 activated Treg (aTreg) and FoxP3 T cells (**Table S2**). An in-depth description of all steps is provided in the **Supplementary Methods**.

## Results

### Characteristics of participants

The baseline characteristics of the inception cohort have been reported previously (11), and those of the random subcohort are shown in **Table S1**. Mothers and infants from this random subcohort who had 16S rRNA gene amplicon sequencing data and who had infant immune measures on either naïve CD4^+^ T cell or Treg cell subsets at birth, 6 or 12 months of age were used for analyses. The definition of complete data for each analysis varied with the timepoint: as shown in **Figure 1**, the proportion of mother-infant dyads with complete data at birth, 6 months and 12 months of birth were 216 of 286 (88%), 181 of 286 (76%) and 219 of 286 (86%), respectively.

### Changes in infant immune cell proportions during the first year of life

For the purposes of this study, immune cell populations in cord blood and at 6 and 12 months as measured by flow cytometry, were assessed as proportions of the total white blood cells (**Supplementary Methods**, **Table S2**). The range of times from collection of blood to processing and staining for flow did not influence the immune cell proportions (*p*=0.6). The medium proportion of granulocytes decreased by almost half (62.1% to 34.9%) from birth to 6 months and then remained stable. This was matched by an overall increase in the lymphocyte subsets, including a doubling in the proportions of CD8^+^ and CD4^+^ T cells (**Table S2**).

### Clusters of co-occurring microbes in maternal fecal samples and relevant exposures

**Figure 3:**
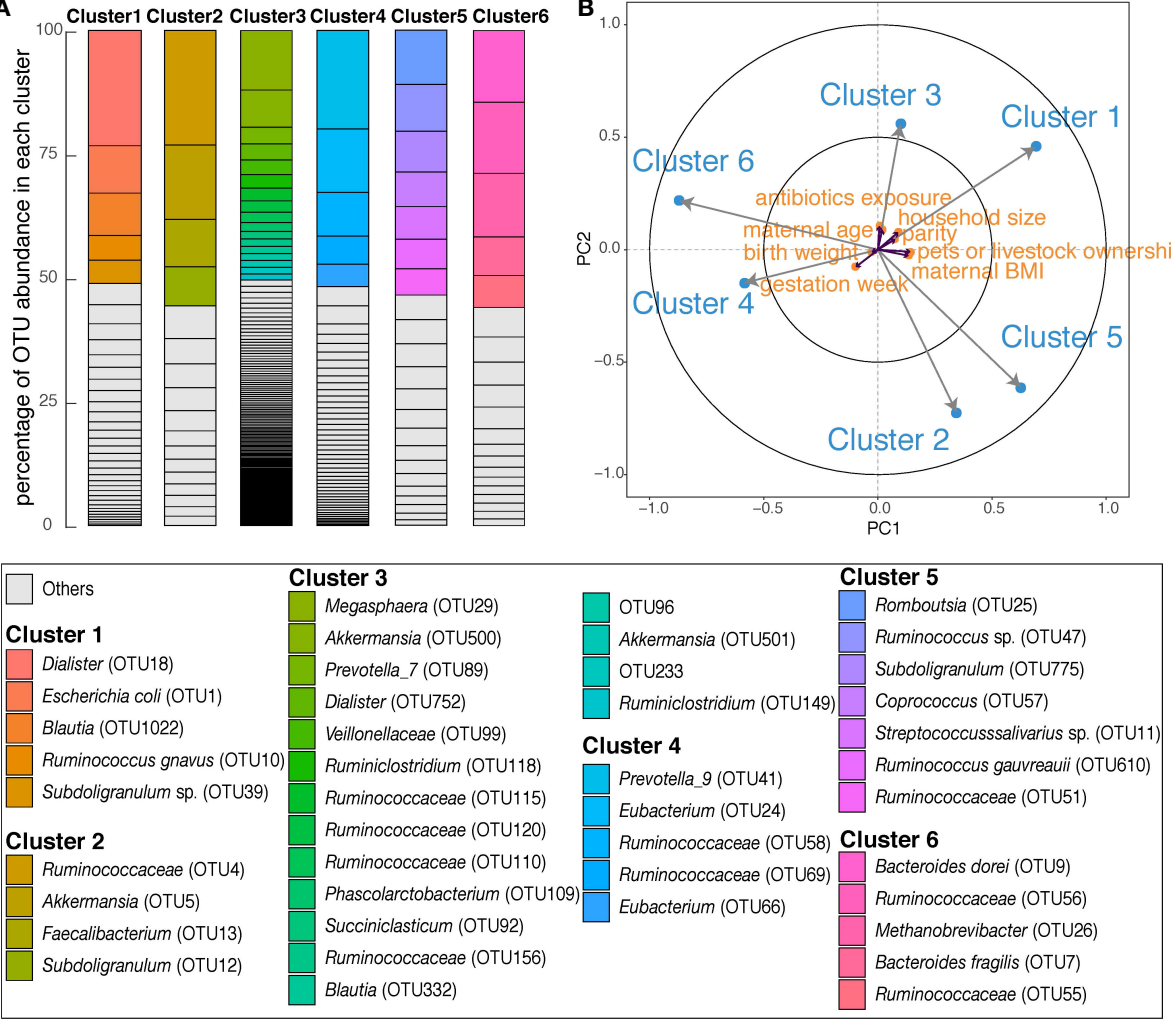
Overall description of six OTU clusters in maternal gut during late pregnancy. **(A)** Each column shows the OTU composition of each cluster. Within each column, OTUs are ordered according to the percentage of their relative abundance in each cluster. The identifications of OTUs on species/genus level are listed in the box below; ‘Others’ are in **Table S3**. **(B)** The partial least squares biplot shows the associations between OTU clusters (blue) and covariates (orange). The longer the arrow the stronger the association with other variables. An acute angle between arrows represents positive association, an obtuse angle represents negative association and orthogonal arrows represent no association.

Six clusters of co-occurring microbes classified by OTU, were identified (**Table S3**). The most abundant taxa in *Cluster 1* were *Dialister*, *Escherichia coli*, *Blautia*, *Ruminococcus gnavus*, and *Subdoligranulum* sp. (**Figure 3A**). These OTUs contributed more than 50% of the total abundance of *Cluster 1*. *Cluster 2* mainly consisted of *Subdoligranulum, Akkermansia*, and *Faecalibacterium.* Most OTUs (*n*=171) were grouped into *Cluster 3*, in which OTUs classified as *Megasphaera, Akkermansia, Prevotella_7, Dialister*, and *Phascolarctobacterium* were most abundant. In *Cluster 4*, *Prevotella_9* was most abundant, followed by *Eubacterium* and *Ruminococcaceae*(**Figure 3A**). *Cluster 5* was composed of Firmicutes only, with *Romboutsia*, *Ruminococcus* sp., *Subdoligranulum, Coprococcus*, and *Streptococcaceae* prominent. The most abundant taxa in *Cluster 6* were *Bacteroides dorei*, *Ruminococcaceae*, *Methanobrevibacter*, and *Bacteroides fragilis*.

We performed PLS regression to explore associations between exposures (maternal age, antibiotics exposure, parity, gestation week, BMI, infant birth weight, household size and pet or livestock ownership) and maternal OTU clusters. Within the PLS model, larger household size associated with *Cluster 1*, and maternal BMI was negatively associated with *Cluster 6* (**Figure 3B**).

### Maternal OTU clusters and immune cell populations in cord blood

**Figure 4:**
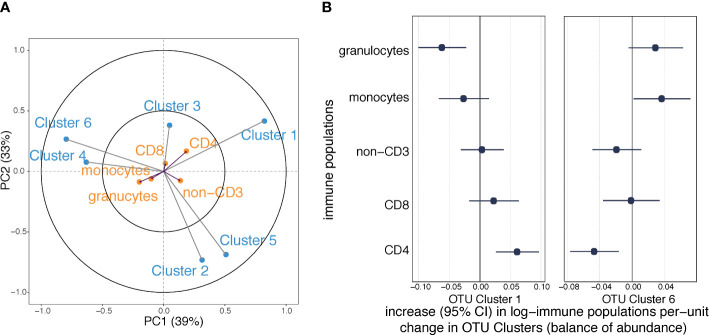
Associations between maternal OTU clusters and immune cell profile in cord blood (*n*=216). **(A)** The partial least squares biplot shows the associations between OTU clusters (blue) and immune populations (orange). The acute angle between arrows to *Cluster 1* and central naïve CD4+ T cells indicates a positive association, whereas the obtuse angle between *Cluster 1* and granulocytes indicates a negative association. **(B)** Increases (with 95% confidence intervals) in log-transformed immune populations per unit change in automated balances for OTU *Cluster 1* and *6* abundances.

We applied a PLS regression model to investigate the covariance between the six maternal OTU clusters and the five offspring immune cell types (granulocytes, monocytes, non-CD3^+^, CD8^+^ and CD4^+^ T cells) measured in cord blood (**Table S2**). The first components of the PLS model explained 39% of the shared OTU-immune covariance and the second component explained 33% (**Figure 4**). Exposure to labor and household size best explained the first two components of OTU-immune PLS space (**Table S4**). Granulocytes and CD4^+^ T cells had longer arrows within the PLS space than the other three immune measures, indicating that they contributed more substantially to the OTU-immune covariance. *Cluster 1* was associated with a lower proportion of granulocytes and a higher proportion of CD4^+^ T cells (**Figure 4A**). *Cluster 6* was associated with a higher proportion of monocytes and a lower proportion of central naïve CD4^+^ T cells (**Figure 4A**).

We then regressed the proportions of the various white cell populations on the OTU clusters. Consistent with the PLS model, maternal carriage of *Cluster 1* predicted a lower proportion of granulocytes and a higher proportion of CD4^+^ T cells (**Figure 4B**); and maternal carriage of *Cluster 6* was associated with lower proportion of granulocytes and higher proportion of monocytes (**Figure 4B**). However, the association between *Cluster* 6 and monocytes was attenuated after adjustment for BMI (**Table S5C**). None of the other examined confounding factors altered the point estimate by greater than 10% (**Table S5**). There was no evidence of other relationships between OTU clusters and immune populations at birth (**Figure S3**).

We performed sensitivity analysis to investigate whether specific microbes might be driving the observed associations. Sequential exclusion of each of the five most abundant OTUs in *Cluster 1* had minimal impact on the evidence that *Cluster 1* was associated with decreased granulocytes and increased CD4^+^ T cells (**Figure S4**), indicating no one specific OTU was driving the association. Similarly, sequential exclusion of each of the five most abundant OTUs in *Cluster 6* had minimal impact of the evidence that *Cluster 6* was associated with lower CD4^+^ T cells (**Figure S5**).

**Figure 5:**
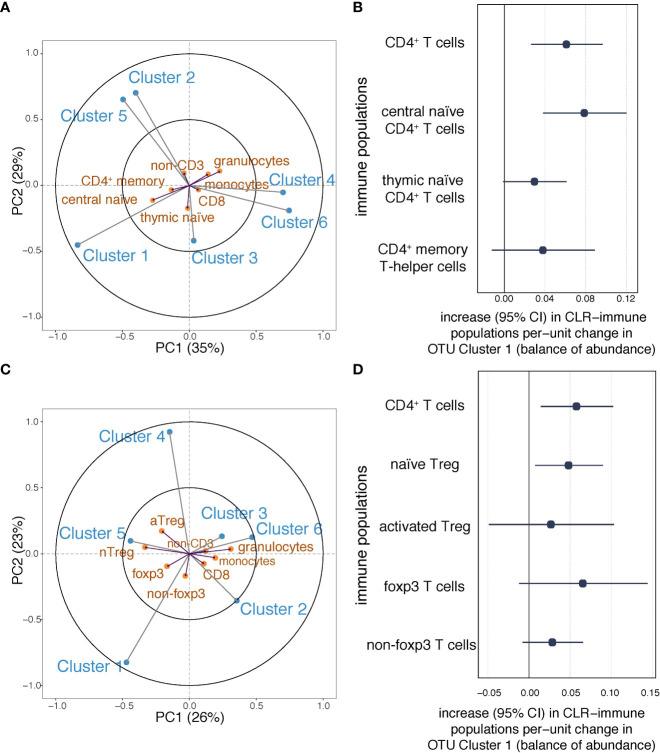
Associations between maternal OTU clusters and CD4+ subpopulations at birth. **(A)** The partial least squares biplot shows the associations between OTU clusters (blue) and immune populations (orange)(*n*=216). The acute angles between *Cluster 1* and central naïve CD4+ T cells suggest a positive association. **(B)** Increases (with 95% confidence intervals) in log-transformed immune populations (CD4+ T cells, and naïve and memory CD4+ T cells) per unit change in automated balances for *Cluster 1* abundance. **(C)** The partial least squares biplot shows the associations between OTU clusters (blue) and immune populations (orange), The acute angle between arrows to *Cluster 1* and naïve Tregs indicates a positive association (*n*=107). **(D)** Increases (with 95% confidence intervals) in log-transformed immune populations (CD4+ T cells, Tregs, and Foxp3 T cells) per unit change in automated balances for *Cluster 1* abundance.

Having observed associations between maternal OTU clusters and total CD4^+^ T cells in the neonate, we investigated covariance between OTU clusters and CD4^+^ T cell subpopulations (**Figure 5A**, **Table S2**). The first component of the PLS model explained 35% of the OTU-immune covariance and the second explained 29% (**Figure 5A**). OTU *Cluster 1* was associated with a higher proportion of each of the CD4^+^ T cell populations, the strongest association being with central naïve CD4^+^ T cells (CD4^+^/CD45RA^+^/CD31^−^) (**Figure 5A**). Conversely, *Cluster 6* was negatively associated with central naïve CD4^+^ T cells. Consistent with the PLS model, in linear regression analyses, *Cluster 1* showed the strongest association with central naïve CD4^+^ T cells (**Figure 5B**). Similar but less compelling evidence was seen for other CD4^+^ sub-populations (thymic naïve CD4^+^ T cells and memory CD4^+^ T cells; **Figure 5B**). *Cluster 6* was associated with decreased central naïve CD4^+^ T cells and thymic naïve CD4^+^ T cells (**Figure S6A**). The association between *Cluster 6* and thymic naïve CD4^+^ T cells was attenuated following adjustment for maternal BMI (**Table S6C**). Adjustment for other potential confounding factors made less than 10% difference to the point estimates (**Table S6C**).

In a subgroup with relevant measures (*n*=107), we applied the PLS regression model to investigate the covariance between maternal OTU clusters and Treg populations in cord blood (**Figure 5C**, **Table S2**). The first component of the PLS model explained 26% of the shared OTU-immune covariance and the second explained 23% (**Figure 5C**). Greater isometric-log-ratio-transformed abundance of OTU *Cluster 1* was associated with a higher proportion of nTreg in cord blood white cells (**Figure 5C**). Linear regression was then used to confirm that OTU *Cluster 1* positively associated with CD4^+^ and nTreg (**Figure 5D**). Following adjustment for household size, the evidence of an association between *Cluster 1* and nTreg at birth was partially attenuated by household size (**Table S6D**), but as household size may impact infant Treg *via* the maternal gut microbiome (11), it is debatable whether such adjustment is appropriate. There was no evidence of an association between *Cluster 6* and either CD4^+^ T cells or Treg populations (**Figure S6B**).

### The role of SCFAs

We investigated the relevance of SCFAs to the observed associations. Higher concentrations of acetate, butyrate and propionate in maternal serum were each associated with a higher proportion of central naïve T cells in cord blood (**Figure S7A**) but not with the proportion of nTreg. OTU *Cluster 1* was associated with higher maternal serum butyrate, but not acetate or propionate (**Figure S7B**). SCFA concentrations in 36-week stool were not associated with cord blood immune measures (**Figure S7A**) and OTU *Cluster 1* was in fact associated with lower stool SCFAs (**Figure S7B**). The concentrations of SCFAs did not associate with the proportion of granulocytes or monocytes (**Figure S7A**). OTU *Cluster 6* was not associated with the concentrations of SCFAs in either stool or serum (**Figure S7B**).

### Maternal OTU clusters and infant immune populations beyond birth

Finally, we fitted regression models to examine the relationships between the isometric-log-ratio-transformed abundances (automated balance) of OTU *Cluster 1* and *Cluster 6* and immune populations at 6 and 12 months. The positive association observed in cord blood at birth between *Cluster 1* and central naïve CD4^+^ T cells remained evident at 6 months (*β*=0.05, 95%CI=(0.002, 0.11)) (**Table S5**); and less convincingly at 12 months (*β* =0.04, 95%CI=(−0.007, 0.09)) (**Table S6**).

In contrast, the negative association observed at birth between *Cluster 1* and granulocytes was not evident at 6 months (**Table S7**) or 12 months (**Table S8**). Similarly, the associations of *Cluster 1* with CD4^+^ T cells and naïve Treg, and *Cluster 6* with CD4^+^ T cells, previously observed at birth, were not evident at 6 months or 12 months (**Table S9**).

## Discussion

As far as we are aware, this is the first human study to investigate associations between maternal gut microbiota in pregnancy and blood immune cell populations over the course of infancy. We found that an OTU cluster in which *Dialister, Escherichia coli*, *Ruminococcus gnavus* were predominant was associated with a lower proportion of granulocytes and a higher proportion of CD4^+^ T cells at birth, in particular central naïve (CD4^+^/CD45RA^+^/CD31^−^) and naïve Treg (CD4^+^/CD45RA^+^/FoxP3^low^) subsets. A second cluster in which *Bacteroides dorei*, *Ruminococcaceae*, *Methanobrevibacter* and *Bacteroides fragilis* were predominant was associated with a lower proportion of overall CD4^+^ T cells. In each case, sequential exclusion of the most abundant OTUs in the cluster made little difference to the observed associations; a finding consistent with growing evidence that communities of gut organisms are, in general, more biologically important than individual taxa (18).

The co-occurrence of bacteria is driven by their interactions and metabolic co-dependencies (19). Acetate is a key metabolic product of *Dialister* (20, 21), and is also associated with higher abundance of *Ruminococcus* (22). Thus, the co-occurrence of *Dialister* and *Ruminococcus* is likely indicative of a gut microbiome producing relatively high concentrations of acetic acid. As previously shown in mice, acetic acid produced by the maternal gut microbiota plays a key role in driving fetal thymic CD4^+^ T cell development, including CD4^+^ Treg (2). Although we found that higher SCFA concentrations in maternal serum, but not stool, were associated with elevated central naïve CD4 T cells, there was little evidence that this finding was driven by the OTU clusters we identified. SCFAs in serum and stool are highly dynamic and there is a need for human studies with repeated measures of diet, gut microbiota and SCFA concentrations in both stool and serum over the course of pregnancy.

*Dialister* and *E. coli* in *Cluster 1* produce lipopolysaccharide (LPS) (23, 24). It was shown in mice, that maternal inoculation with a LPS-producing bacterium, *Acinetobacter lwoffii* F78, increased Toll-like receptor (TLR) 2, 3, 6, 7, and 9 mRNA expression in the maternal lung tissue, decreased mRNA expression in the placenta and decreased allergic responses in the offspring (25). *TLR2/3/4/7/9^−/−^* knockout mice were then used to demonstrate that this pathway of fetal immune programming was TLR-dependent; however, LPS was not detected in the amniotic fluid, suggesting the process was not driven by direct exposure of the fetus to LPS (25). Subsequent experiments in germ-free mice have shown that maternal carriage of *E.coli* during pregnancy drives innate immune development in the fetus and that this process is dependent in part on maternal IgG (1). IgG is both passively and actively transported across the placenta and may carry microbiota-derived molecules such as LPS, to stimulate the developing fetal immune system. Whether these findings are relevant to the association we observed between maternal carriage of *Dialister* and *E. coli* and decreased granulocytes in the offspring is uncertain, but the finding is potentially consistent with preclinical evidence of LPS-induced *in utero* innate immune training.

The granulocyte proportion measured here comprises neutrophils plus the minor populations of eosinophils and basophils. Labor is associated with increased neutrophils (26). We have previously reported that exposure to labor may reveal differences in the innate and adaptive immune phenotype among infants who subsequently develop allergic disease (27, 28), and that increased innate immune (CD14+ monocyte) responsiveness at birth predicts subsequent allergic disease (27, 29). Although granulocytes play a crucial role in innate immune responses, they are difficult to work with and remain strikingly understudied. The potential relevance of early life granulocyte ontogeny and function to immune-mediated and infectious diseases presents an important challenge and opportunity for future research.

At birth the majority of CD4^+^ T cells have a naïve phenotype (12, 26) reflecting the relative absence of antigenic stimulation *in utero*. Naïve CD4^+^ T cells comprise two subsets, differentiated by the marker CD31, central naïve and thymic naïve (30). CD31 is involved in modulation of T-cell receptor (TCR) signaling (31) and the CD31 negative central naïve CD4^+^ T cells represent the cell pool capable of undergoing homeostatic proliferation (32). The transition from thymic naïve to proliferating central naïve CD4^+^ T cell is initiated *via* the interaction of the T cell receptor (TCR) with major histocompatibility complex (MHC) class II–presented peptides, many of which are believed to be self-antigens (30). Immune-active products of gut microbiota, such as acetate produced by *Dialister* and LPS by *Dialister and E. coli*, could enhance presentation of self-peptides by antigen-presenting cells (APC), and potentially explain the observed increased proportions of both the naïve CD4^+^ and central naïve CD4^+^ T cells at birth.

A higher proportion of nTreg at birth is associated with deceased risk of subsequent allergic disease (27, 33, 34). In this context, the association between maternal gut microbiota cluster enriched for *Dialister, E. coli* and *Ruminococcus gnavus* and higher level of nTreg at birth adds to the emerging evidence regarding the role of the maternal gut microbiome in the prevention of allergic disease and asthma in the infant (3), although further studies are required to determine whether this cluster associates with relevant clinical outcomes and to delineate underlying mechanisms.

The clinical relevance of increased proliferating central naïve T cells is less easily defined. It is likely that the CD4^+^/CD45RA^+^/CD31^−^ population we describe is contained within the population described as ‘neonatal T cells’. Rather than simply being precursors of adult T cells, neonatal T cells are a broadly reactive ‘developmental layer’, poised to quickly differentiate into effector or regulatory cells, thus enabling the immunologically naïve infant to mount efficient and regulated responses to the postnatal environment (32). Tregs derived from neonatal T cells are maintained into adulthood and play a key role in preventing systemic autoimmunity (35). It is thus interesting to consider whether the maternal gut microbiome might protect the infant from developing autoimmune disease by promoting neonatal T cells during pregnancy. Infants of mothers with type 1 diabetes (T1D) are substantially less likely to develop T1D than infants of fathers with T1D (36). We have recently shown that in women with T1D the gut microbiome during pregnancy is characterized by an increase in *E. coli* carriage and LPS production capacity (37). Given the association between an *E. coli* dominant cluster and the central naïve CD4+ population observed here, we hypothesize that protective effect of maternal vs. paternal T1D is mediated by an *E. coli* dominant maternal microbiome promoting the development of neonatal T cells in the infant.

The strengths of this study include longitudinal design incorporating the collection of maternal feces during pregnancy, offspring flow cytometry measures at three timepoints from birth through infancy, and detailed consideration of potential confounders. Flow cytometry was conducted on fresh blood, which is important as it allows analysis of granulocytes and removes potential influences from monocyte purification and potential selective loss of subpopulations due to freeze-thaw. By aggregating OTUs into clusters, we derived composite biomarkers that are likely to be more robust to the low read counts inherent to 16S gene amplicon sequencing, and which performed well empirically in predictive modelling (38, 39). Species within the same cluster may share a wide range of biological responses and metabolic capabilities representing a self-contained network; our approach thus provided a high-level view of the association between maternal gut microbiota and offspring immunity.

This study has several limitations. The flow cytometry commenced over a decade ago and included only a small number of cell surface markers to delineate the cell populations, and thus other cell populations were not examined. Given that immune measures are proportional data, the frequencies of the various populations are co-dependent, and the absolute numbers are unknown. The compositional analysis we have employed is however a more appropriate approach than simply investigating proportions within a given subgroup such as CD4^+^ T cells. Our immune measures are non-functional and are limited to the cord and peripheral blood compartments. Human studies including a broader range of cell populations and functional readouts, for example cytokine production in response to various ligands, are required to describe a more complete picture of both the innate and adaptive arms of the infant’s immune system. Within the bacterial clusters, not all taxa have been studied in detail, and the net biological functions and importance of each cluster is unknown. Metagenomic sequencing is required to provide sufficient resolution at the species and strain level to infer functional capacity, combined with analytic strategies that group organisms based on function rather than mere co-existence.

In conclusion, this human cohort study corroborates the growing body of pre-clinical evidence that the maternal gut microbiome plays a key role in driving fetal immune development. Improved understanding of the underlying pathways and their clinical relevance is likely to provide novel targets for the primary prevention of immune-related disorders such as allergy, asthma and T1D, each of which have become substantially more common in the modern environment.

## Data availability statement

The data analyzed in this study is subject to the following licenses/restrictions: Anonymized data used in the analysis, results and conclusions reported in this paper will be made available to any researcher for purposes of reproducing or extending the analysis. Requests to access these datasets should be directed to BIS@BarwonHealth.org.au.

## Ethics statement

The studies involving human participants were reviewed and approved by The study was approved by the Barwon Health Human Research and Ethics Committee (HREC 10/24). Written informed consent to participate in this study was provided by the participants’ legal guardian/next of kin.

## Author contributions

YG and TQ performed the analyses. YG, TQ, MOH, FC and PV wrote the manuscript. PV, FC, MOH, ALP, LH, HF, MT, SB and KK revised the manuscript. PV, FC and MOH supervised the project. All authors read, edited and approved the final manuscript.

## Funding

This study was funded by the National Health and Medical Research Council of Australia (1082307, 1147980), the Australian Food Allergy Foundation, The Murdoch Children’s Research Institute, Barwon Health and Deakin University.

## Acknowledgments

We wish to thank Prof. John Carlin for his advice. We also wish to thank the BIS team for sample collection and all BIS participants.

## Conflict of interest

The findings described in this paper are the subject of a provisional patent, licensed to Prevatex Pty Ltd, in which the following authors have a financial interest: PV, MOH, SR, ALP.

The remaining authors declare that the research was conducted in the absence of any commercial or financial relationships that could be construed as a potential conflict of interest.

## Publisher’s note

All claims expressed in this article are solely those of the authors and do not necessarily represent those of their affiliated organizations, or those of the publisher, the editors and the reviewers. Any product that may be evaluated in this article, or claim that may be made by its manufacturer, is not guaranteed or endorsed by the publisher.
