# Supplementary material for: Maternal gut microbiota during pregnancy and the composition of immune cells in infancy
Source: Front Immunol. 2022 Sep 21;13:986340. doi: 10.3389/fimmu.2022.986340 (PMC9535361; doi:10.3389/fimmu.2022.986340)
Supplement: Supplementary file 1 [file DataSheet_1.docx]

# SUPPLEMENTARY MATERIAL

## SUPPLEMENTARY METHODS SUPPLEMENTARY FIGURE CAPTIONS

**SUPPLEMENTARY METHODS**

## Dimensionality reduction of the OTUs

We assessed clusters for both biological and technical components of variation. First, we hypothesized that the correlated bacteria species may occupy a “functional niche” that together impart an influence on the offspring *immune profile*. By aggregating OTUs into clusters, we defined data-driven “functional niches” that allowed us to directly test for associations between them and the *immune profile*. Second, cluster-level measurements are more stable than taxon-level measurements, whereby individual taxa can be “missed” due to under-sequencing ^1^. Third, high-throughput assays recorded abundances for many OTUs. By aggregating OTUs into clusters, we reduced the total number of statistical tests needed, which served to improve power of the study overall.

Using prevalence filtering, we selected the 323 out of 1628 OTUs whose per-OTU sum exceeded 0.01% of the total sum across all rows and columns^2^. In *k*-means clustering, we grouped bacteria into *clusters* based on how they correlated across the subjects. Specifically, in viewing the OTU abundances as compositional data (c.f.,^3-5^), we performed a centred log-ratio (CLR) transformation on the data^6^ after offsetting all abundances by 1 (to avoid taking the log of zero). We clustered the OTUs using *k*-means clustering of the Euclidean distances among the columns (i.e., OTUs) of the CLR-transformed^7^ samples-by-OTUs table. We assessed the optimal number of clusters by applying Gap Statistic method using R package *factoextra and* opted to use 6 clusters (Figure S8). It should be emphasised that these are clusters of OTUs and not of samples/participants. The clusters are more similar to microbial co-abundance groups^8^ than enterotypes.^2^ Individuals are characterised by the overall abundances of the OTU clusters within their microbiota, rather than being assigned to a given enterotype.

Once the clusters were defined using *k*-means (*k*=6), the raw (untransformed) OTU counts were added together (called amalgamation c.f.,^9^), then CLR-transformed. No samples had any zeroes after addition, so no offsetting was performed. These OTU clusters formed the *microbiome signature* dataset.

## Pre-processing of infant immune profiling data

Table S2 shows the immune cell populations measured by flow cytometry and assessed as proportions of the total white cells. Analyses were performed initially for subsets of granulocytes (gos), monocytes (mos), CD3 negative lymphocytes (CD3-), CD8^+^ cytotoxic T cells (CD8), CD4^+^ T helper cells (CD4). Secondary analyses were performed on sub-populations of the CD4^+^ T cells. These comprised naive subsets; central naïve CD4^+^ T cells (q1), thymic naïve CD4^+^ T cells (q2) and CD4^+^ memory T-helper cells (q3), and Treg subsets with proportions of FoxP3 naïve Treg (nTreg), FoxP3 activated Treg (aTreg) and FoxP3 T cells. The median percentage of the total white cells (with IQR) is presented for each population. These variables represent fractions of the whole, and so roughly sum to 100% of the immune cell population. Like the OTUs, we standardised the data using the CLR transformation, enabling us to use the double-centred multi-omics transformation ^10^.

## Multivariable ordination analysis

Partial least squares (PLS) regression^11^ is an ordination method conceptually related to principal components analysis (PCA). Like PCA, PLS finds new variables, called *components*, which represent the structure within the data sets. In PCA, these components recursively maximise the variance within a single data set. In PLS, these components maximise the covariance between two data sets. Herein, we performed PLS using the mixOmics package in R^12^. In our case, the two data sets were either the microbiome signature and clinical covariates, or the microbiome signature and *immune profile* datasets. The first two components, capturing most of the covariance, can provide a two-dimensional visualization of the multi-omic association.

## Forward stepwise selection of clinical covariates

We selected a subset of covariates that best predict the associations identified by PLS regression model by Akaike information criterion (AIC) in a stepwise algorithm (*step* in R). Covariates considered by the *step* algorithm were maternal age, BMI, intake of antibiotics, parity, household size, ownership of pets or livestock, birth gestation week, infant sex, exposure to labour, birth weight, and feeding practice. Exposure to labour was categorised according to either (a) Absence of labour: infants born by Caesarean section (C-section) prior to the onset of labour, or (b) Exposure to labour: remaining infants, including those born by C-section following the onset of labour (Table S1). For each of the first two components of PLS regression, we started with a null model as the baseline model and added each one of the 11 covariates to identify the one that yields the smallest AIC. Using this covariate as the new baseline model, we then added each one of the 10 remaining covariates again, and so on until the step algorithm converged on the smallest AIC.

**Automated balances**

Although the CLR is popular in microbiome data analysis, the interpretation of the coefficient of a CLR component can be problematic because of a constraint that forces the CLR components to sum to zero^13^. As such, a CLR component reflects the rate of change of the outcome relative to a change in the component in question, while the remaining estimated components are held constant and an omitted component undergoes an equal and opposite change. Balances provide another approach to compositional data analysis in a way that removes the constraint. There are many ways to construct balances; we use *automated balances* as described by Kynčlová *et al.*^14^.

Balances are a set of interpretable log-contrasts, produced by an isometric log-ratio (ILR) transformation^15,16^, often used to analyze microbiome data^17-21^. Automated balances can be defined as follows. Let $x_{ij}$ be the relative abundance of OTU cluster $j$ in sample $i$ and let $D$ be the total number of clusters represented by the composition, so in our case $D=6$. In analysing OTU cluster *k*, the first automated balance

$$z_{ik}=\sqrt{\frac{D-1}{D}}\text{log}\frac{x_{ik}}{\sqrt[D-1]{\prod_{j\in\left\{ 1,\ldots,D \right\}\left\{ k \right\}} x_{ij}}}$$

is of interest. We call $z_{ik}$ the $k$*-primary balance*. It describes the dominance of the OTU cluster $k$ as compared with all other clusters (30). The remaining *auxiliary* balances recursively describe the dominance of each other cluster, to all those not yet separately considered. Figure S9 and Table S10 present an illustration of automated balances where OTU cluster 4 is of interest. When using automated balances as linear predictors in a multivariable regression, the coefficient of the $k$-primary balance describes the effect of varying the abundance of OTU cluster $k$ while keeping the abundances of the other clusters in fixed proportion to each other. We emphasise that when analysing each of the six OTU clusters, we generate and regress on a different set of automated balances and only interpret the regression coefficient corresponding to the respective primary balance.

In Figure S9 and Table S10, *Cluster 4* is in the first row and so *Cluster 4* is the balance-of-interest. Setting *Cluster 1* as the first row will make *Cluster 1* the balance-of-interest, and so on. (The remaining columns of the automated balances yield an additional $D-2$ balances that describe how the remaining OTU clusters vary as compared with one another. They are less interpretable but are used to adjust a model that includes the balance-of-interest as a predictor. They guarantee that, when performing a multivariable regression of some outcome on the balances, the coefficient estimated for the balance-of-interest represents the modelled change in the outcome per change in the balance-of-interest, with the remaining CLR co-ordinates staying in fixed proportion to each other.)

## OTU cluster balances for linear regressions

### Regressing balances against clinical covariates

To test the relationship between a clinical covariate and an OTU cluster, we fitted a linear regression model for the covariate as a function of the primary balance for the cluster-of-interest and its associated auxiliary balances. In this model, one can interpret the coefficient for the primary balance as the contribution of that OTU cluster to the prediction of the clinical covariate, where the coefficient (*γ*) reflects the change of ratio of the given cluster's abundance, to the geometric mean of the remaining clusters' abundances, by one unit on the log scale. Separate models were used for each cluster. To investigate whether individual OTUs drive the association between OTU *Cluster 1* and clinical covariates, we performed a *sensitivity analysis* where we fit additional regression models such that the prominent OTUs from the cluster-of-interest were removed.

### Regressing balances against *immune profile*

Similarly, to examine relationships between immune cell type abundance and an OTU cluster, we fitted a linear regression to the (CLR-transformed) immune cell proportions as a function of the primary balance for the cluster, its associated auxiliary balances, and selected covariates. This was performed for each of the *immune profile* measures including granulocytes, monocytes, CD3^-^, CD8^+^, and CD4^+^ T cells and its subpopulations at each of the 3 timepoints (Table S2). To examine whether individual OTUs drive the association between an OTU cluster and the *immune profile*, we analysed additional regression models, where each had one of the most prominent OTUs removed from the entire dataset.

### Potential confounding factors in linear regressions.

Potential confounding factors, including sex, household size, maternal age, maternal BMI, pet or livestock ownership, and use of antibiotics during the third trimester were selected based on the disjunctive cause criterion^22^ and those changed estimate by more than 10% were included in the regressions (Table S5, S6, S11). We then divided samples based on tertiles of the cluster abundance and examined the estimates across three groups to avoid antecedents. For example, among these covariates, adding maternal BMI into the regression model against monocytes decreased the estimate of *Cluster 6* by more than 10% (*β*=0.02) (Table S5c). After samples were divided based on tertiles of the abundance of *Cluster 6*, the association between maternal BMI and monocytes were attenuated in all groups, suggesting that maternal age was associated with monocytes independent from *Cluster 6*. We then included maternal BMI into the confounding list. Similarly, we considered maternal BMI in regressing *Cluster 6* against neonate thymic naïve CD4^+^ T cells (Table S6c), and household size in regression Cluster 1 against nTreg (Table S6d).

**SUPPLEMENTARY FIGURE CAPTIONS**

**Figure S1.** **The distribution of 286 mother-infant pairs (including 2 pairs of twins) at three time points.** The analyses were based on mother-infant pairs had both maternal fecal 16S sequence data and adequate infant immune measures from at least one of birth, 6 and 12 months.

**Figure S2.** **Gating of flow cytometry data to identify immune cell subsets.** Whole blood cells were surface stained with fluorochrome-labelled monoclonal antibodies to human CD3, CD4 and CD45. The granulocyte (gos), monocytes (mos) and lymphocytes (los) **(A)** were gated on the basis of CD45 positivity and granularity (side scatter, SSC). T cell populations (CD4, CD8, and non-CD3) **(B)** were gated on the basis of CD3 and CD4 expression. The naïve CD4^+^ T cell populations **(C)** were gated according to CD4 positivity and staining by human antibodies to CD45RA and CD31. Central naïve CD4^+^ T cells (q1) were defined as CD4^+^CD45RA^+^CD31^-^, the thymic naïve CD4^+^ T cells (q2) were CD4^+^CD45RA^+^CD31^+^, and CD4^+^ memory T-helper cells were CD4^+^CD45RA^-^CD31^-^ (q3). The CD4^+^ regulatory T cell (Treg) populations **(D)** were gated according to CD4 positivity and staining by human antibodies to CD45RA and FoxP3. Naïve/resting Treg (nTreg) were classified as CD45RA^+^FoxP3^low^, activated Treg (aTreg) as CD45RA^-^FoxP3^high^, and non-suppressive FoxP3 T cells (foxp3tcells) as CD45RA^-^FoxP3^low^.

**Figure S3. The associations between maternal OTU Clusters 2,3,4,5, and five immune populations in cord blood detected by regression models.**

**Figure S4. The associations between maternal OTU Cluster 1 and granulocytes (left panel), as well as CD4^+^ T cells (right panel) in cord blood detected by regression models**, with each of the five most abundant OTUs in Cluster 1 excluded at a time.

**Figure S5. The associations between maternal OTU Cluster 6 and CD4^+^ T cells in cord blood detected by regression models**, with each of the five most abundant OTUs in Cluster 6 excluded at a time.

**Figure S6. The associations between maternal OTU Cluster 6, and and subpopulations of CD4^+^ T cells in cord blood.** **(A)** Increases (with 95% confidence intervals) in log-transformed immune populations (naïve CD4^+^ T cells, thymic CD4^+^ T cells and memory T cells) per unit change in automated balances for Cluster 6 abundance (n=216). **(B)** Increases (with 95% confidence intervals) in log-transformed immune populations (neonatal CD4^+^ T cells and its subpopulations including naïve Treg, activated Treg, foxp3 T cells and non-foxp3 T cells) per unit change in automated balances for Cluster 6 abundance (n=107).

**Figure S7.** **The role of SCFAs in the OTU-immune associations.** **(A)** Each horizontal line represents an immune phenotype in cord blood. Dots represent the point estimate for immune phenotypes and lines show the 95% confidence interval per 1-SD increment in SCFA acetate, propionate and butyrate. **(B)** Dots represent the point estimate for SCFAs and lines show the 95% confidence interval per 1-SD increment in maternal Cluster 1 and Cluster 6.

**Figure S8. The optimal number of maternal clusters as defined by Gap-static Method.** The optimal number is indicated by dash lines.

**Figure S9. An illustration of automated balances.** In this example OTU Cluster 4 is of interest.

**References**

1 Silverman, J. D., Roche, K., Mukherjee, S. & David, L. A. Naught all zeros in sequence count data are the same. *Computational and structural biotechnology journal* **18**, 2789 (2020).

2 Consortium, M. *et al.* Enterotypes of the human gut microbiome. *Nature* **473**, 174-180 (2011).

3 Gloor, G. B., Wu, J. R., Pawlowsky-Glahn, V. & Egozcue, J. J. It's all relative: analyzing microbiome data as compositions. *Annals of Epidemiology* **26**, 322-329 (2016).

4 Gloor, G. B., Macklaim, J. M., Pawlowsky-Glahn, V. & Egozcue, J. J. Microbiome Datasets Are Compositional: And This Is Not Optional. *Frontiers in Microbiology* **8**, 2224 (2017).

5 Quinn, T. P., Erb, I., Richardson, M. F. & Crowley, T. M. Understanding sequencing data as compositions: an outlook and review. *Bioinformatics* **34**, 2870-2878 (2018).

6 Aitchison, J. *The Statistical Analysis of Compositional Data*. (Springer Netherlands, 1986).

7 Aitchison, J., Barceló-Vidal, C., Martín-Fernández, J. A. & Pawlowsky-Glahn, V. Logratio Analysis and Compositional Distance. *Mathematical Geology* **32**, 271-275 (2000).

8 Jha, A. R. *et al.* Gut microbiome transition across a lifestyle gradient in Himalaya. *PLoS biology* **16**, e2005396 (2018).

9 Greenacre, M. Amalgamations are valid in compositional data analysis, can be used in agglomerative clustering, and their logratios have an inverse transformation. *Applied Computing and Geosciences* **5**, 100017 (2020).

10 Quinn, T. P. & Erb, I. Examining microbe–metabolite correlations by linear methods. *Nature Methods* **18**, 37-39 (2021).

11 Abdi, H. Partial least squares regression and projection on latent structure regression (PLS Regression). *WIREs Computational Statistics* **2**, 97-106 (2010).

12 mixOmics (Bioconductor, 2018).

13 Boogaart, K. G. v. d. & Tolosana-Delgado, R. *Introduction. Analyzing compositional data with R*. (Springer, 2013).

14 Kynčlová, P., Hron, K. & Filzmoser, P. Correlation Between Compositional Parts Based on Symmetric Balances. *Mathematical Geosciences* **49**, 777-796 (2017).

15 Egozcue, J. J. Isometric Logratio Transformations for Compositional Data Analysis. *Mathematical Geology* **35**, 279-300 (2003).

16 Egozcue, J. J. & Pawlowsky-Glahn, V. Groups of Parts and Their Balances in Compositional Data Analysis. *Mathematical Geology* **37**, 795-828 (2005).

17 Rivera-Pinto, J. *et al.* Balances: a New Perspective for Microbiome Analysis. *mSystems* **3**, e00053-00018, /msystems/00053/00054/msys.00053-00018.atom (2018).

18 Morton, J. T. *et al.* Balance Trees Reveal Microbial Niche Differentiation. *mSystems* **2**, mSystems.00162-00116, e00162-00116 (2017).

19 Silverman, J. D., Washburne, A. D., Mukherjee, S. & David, L. A. A phylogenetic transform enhances analysis of compositional microbiota data. *eLife* **6**, e21887 (2017).

20 Washburne, A. D. *et al.* Phylogenetic factorization of compositional data yields lineage-level associations in microbiome datasets. *PeerJ* **5**, e2969 (2017).

21 Quinn, T. P. & Erb, I. Interpretable Log Contrasts for the Classification of Health Biomarkers: a New Approach to Balance Selection. *mSystems* **5**, e00230-00219, /msystems/00235/00232/msys.00230-00219.atom (2020).

22 VanderWeele, T. J. Principles of confounder selection. *European journal of epidemiology* **34**, 211-219 (2019).
