# Supplementary figures and images for: Maternal gut microbiota during pregnancy and the composition of immune cells in infancy
Source: Front Immunol. 2022 Sep 21;13:986340. doi: 10.3389/fimmu.2022.986340 (PMC9535361; doi:10.3389/fimmu.2022.986340)

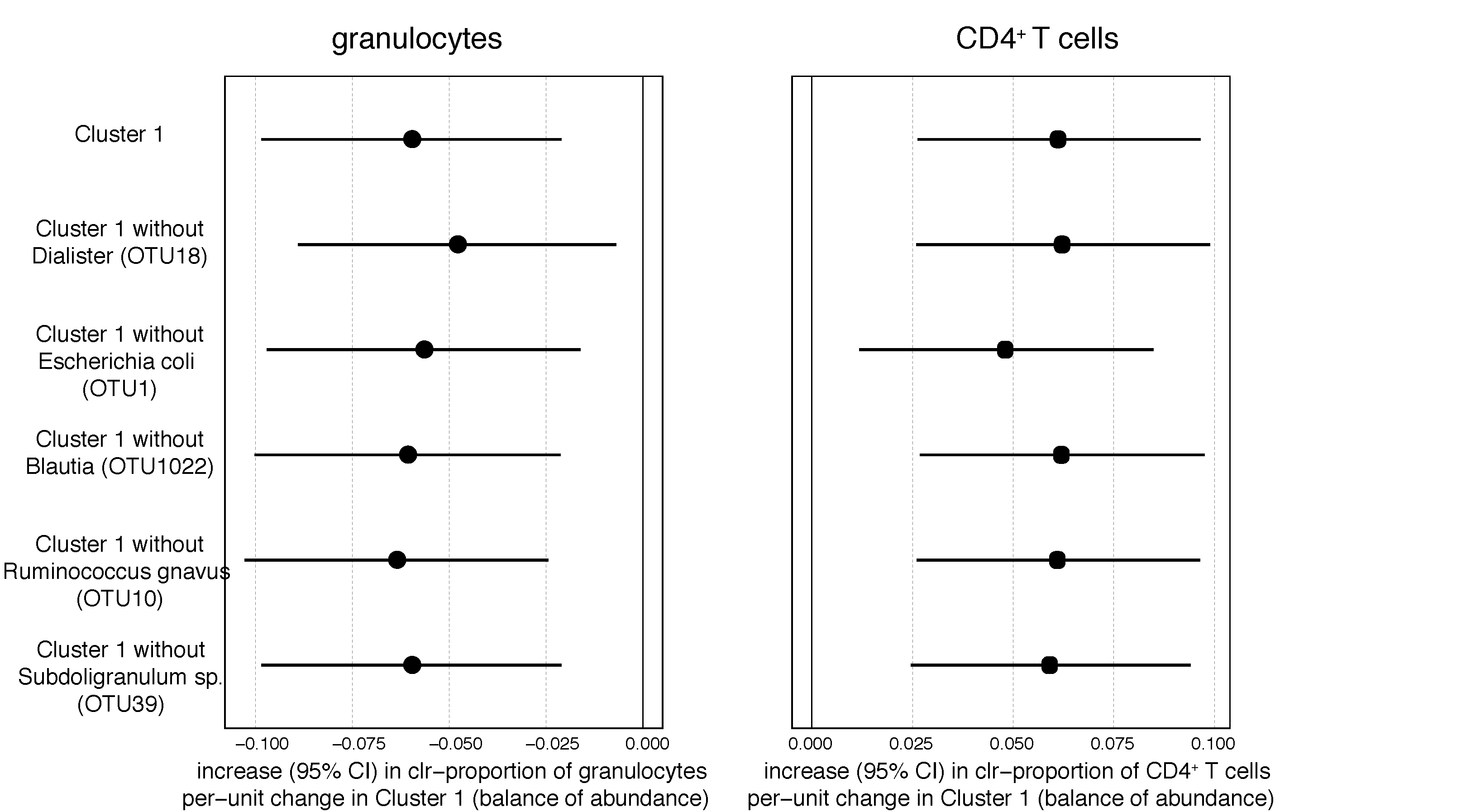

Supplement: Supplementary file 3 [file DataSheet_3.zip › Supplementary Material Presentation/supp. figure 4.png]

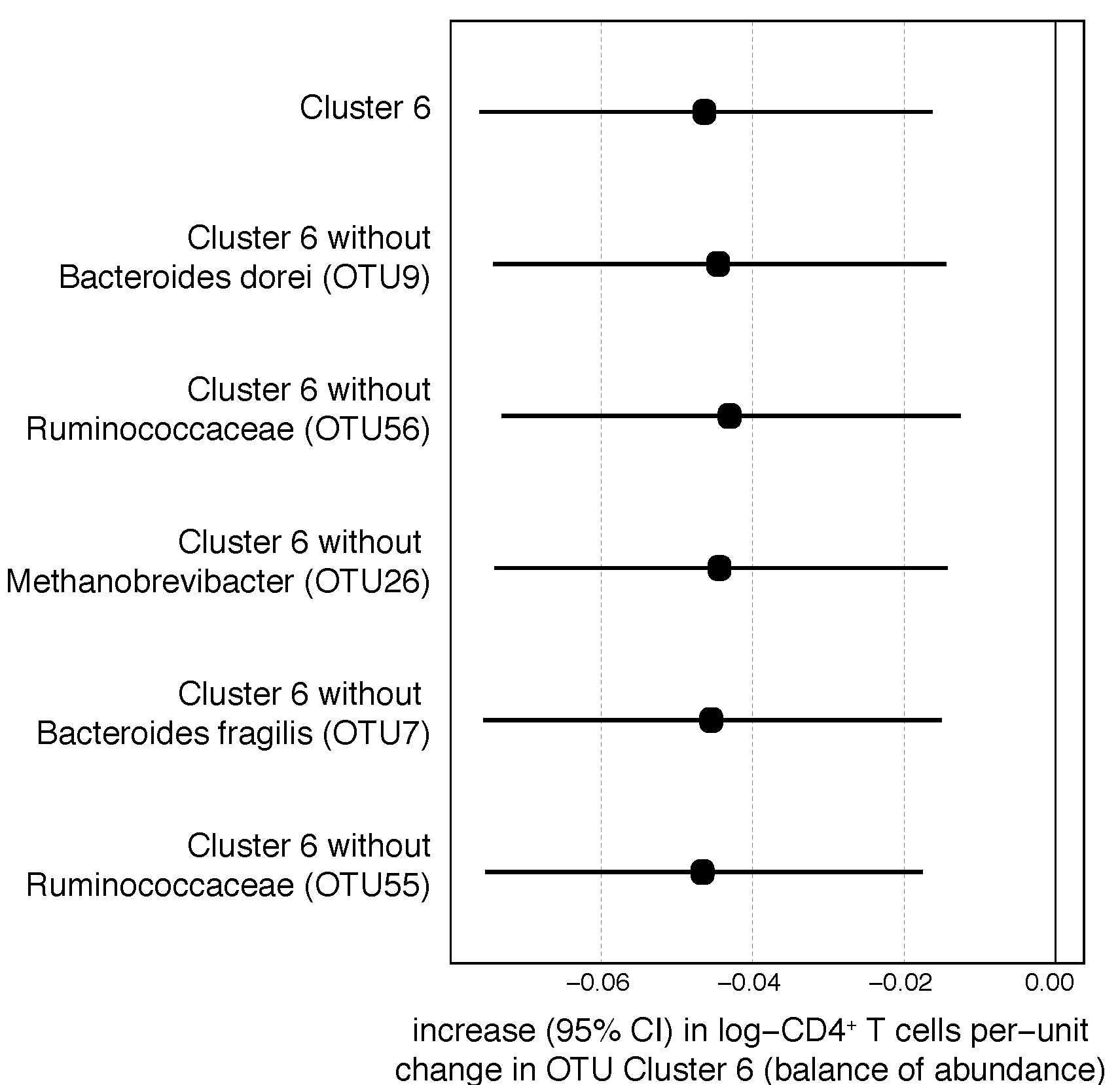

Supplement: Supplementary file 3 [file DataSheet_3.zip › Supplementary Material Presentation/supp. figure 5.png]

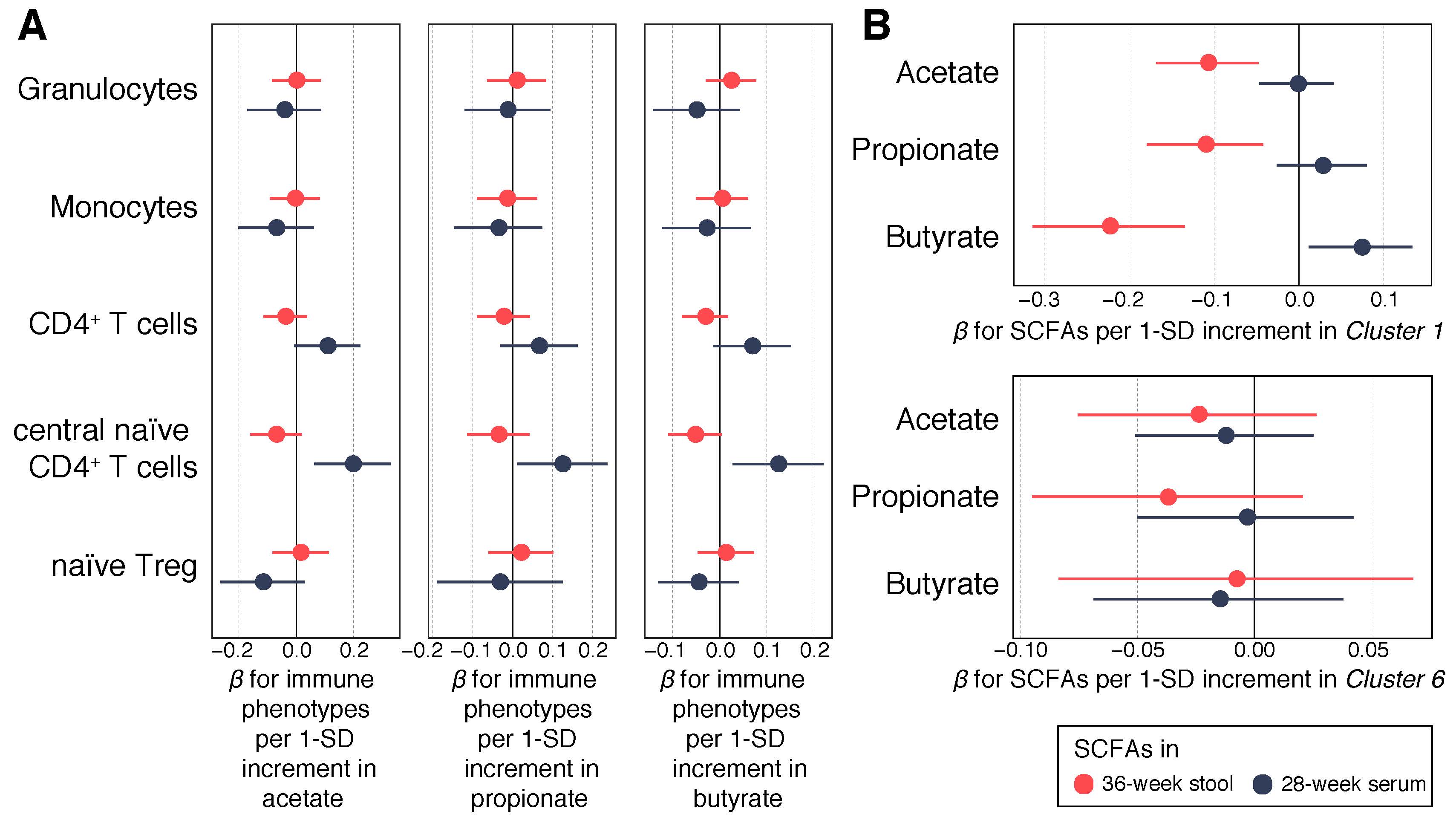

Supplement: Supplementary file 3 [file DataSheet_3.zip › Supplementary Material Presentation/supp. figure 7.jpg]

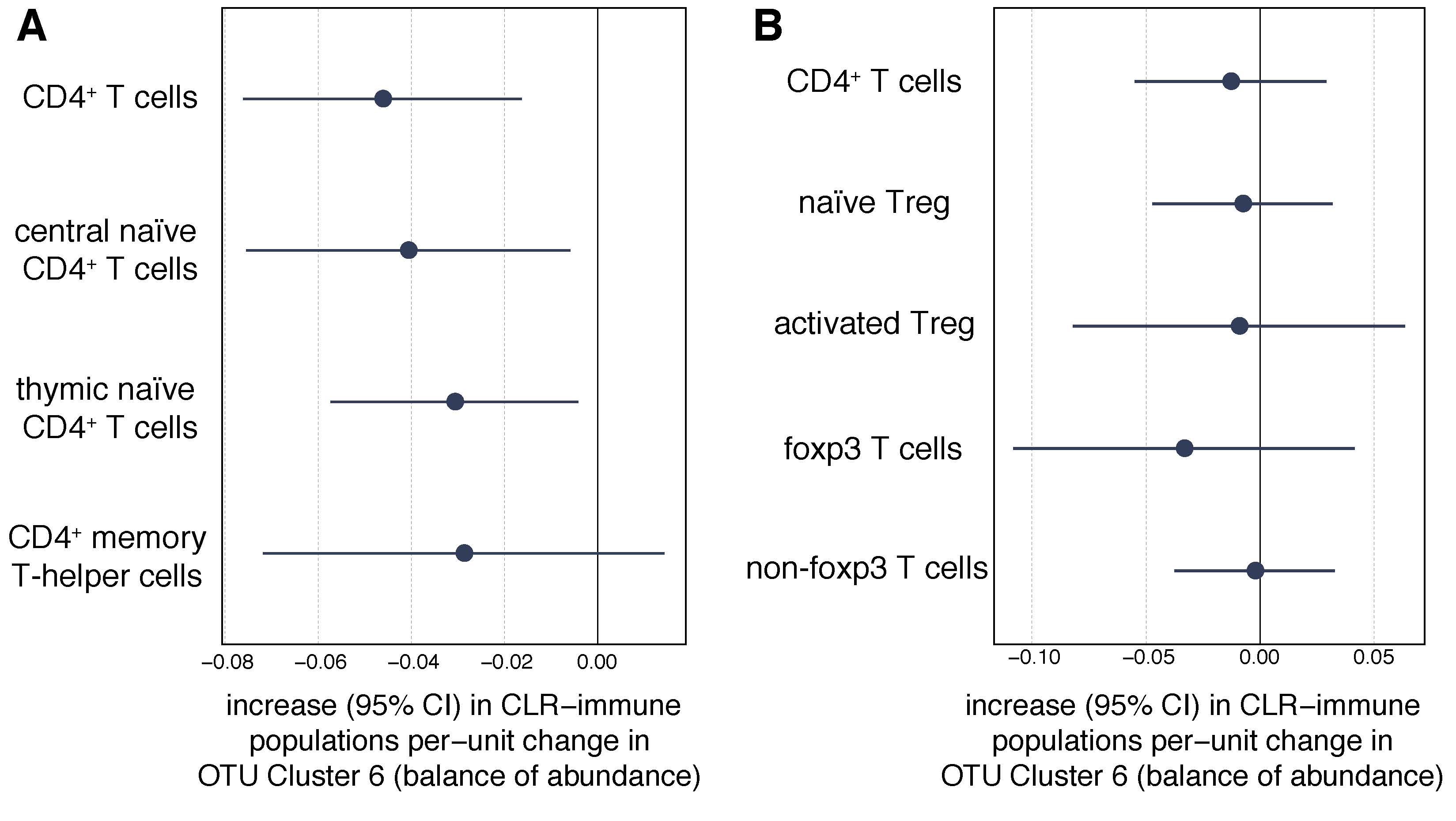

Supplement: Supplementary file 3 [file DataSheet_3.zip › Supplementary Material Presentation/supp. figure 6.jpg]

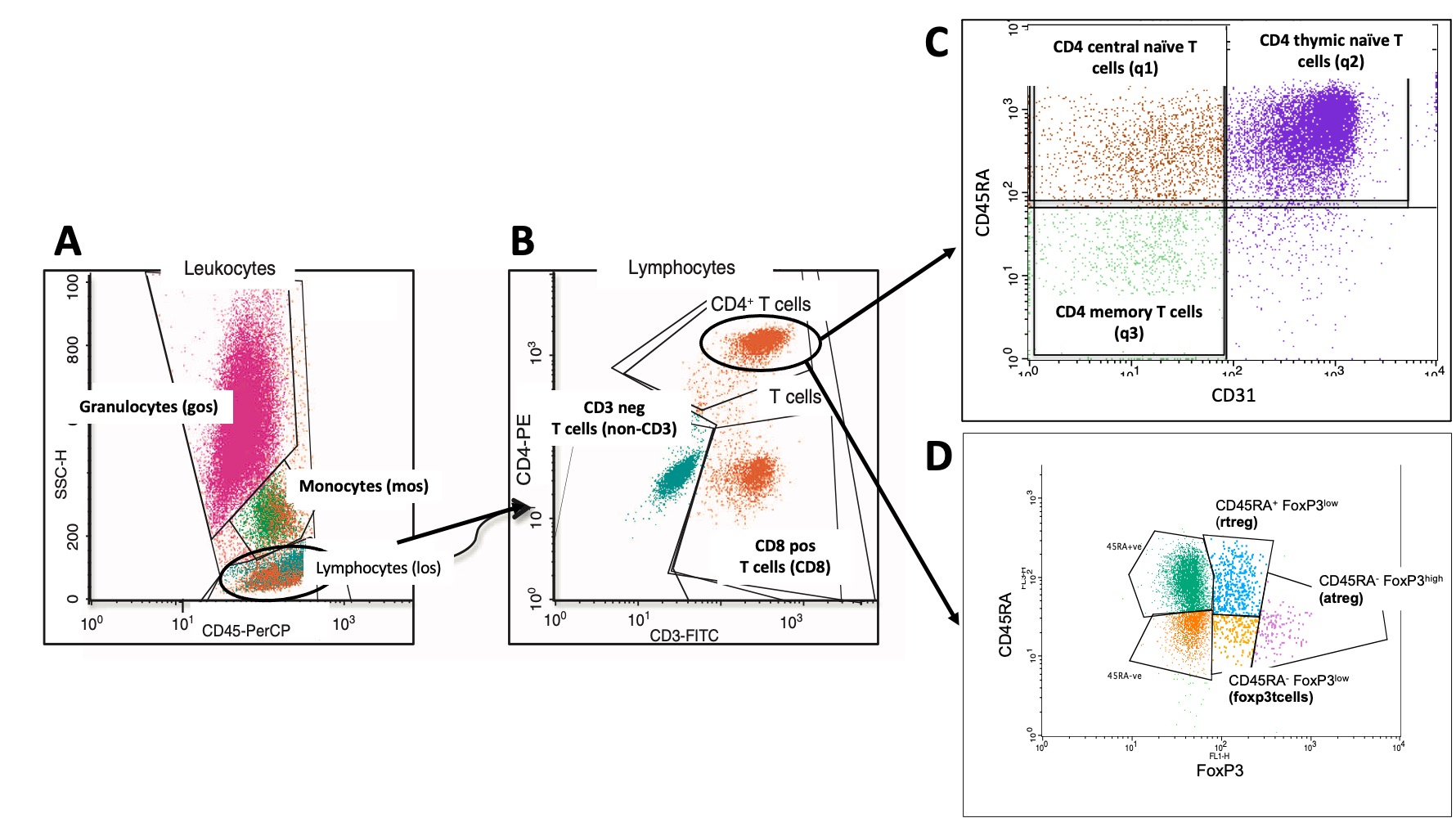

Supplement: Supplementary file 3 [file DataSheet_3.zip › Supplementary Material Presentation/supp. figure 2.jpg]

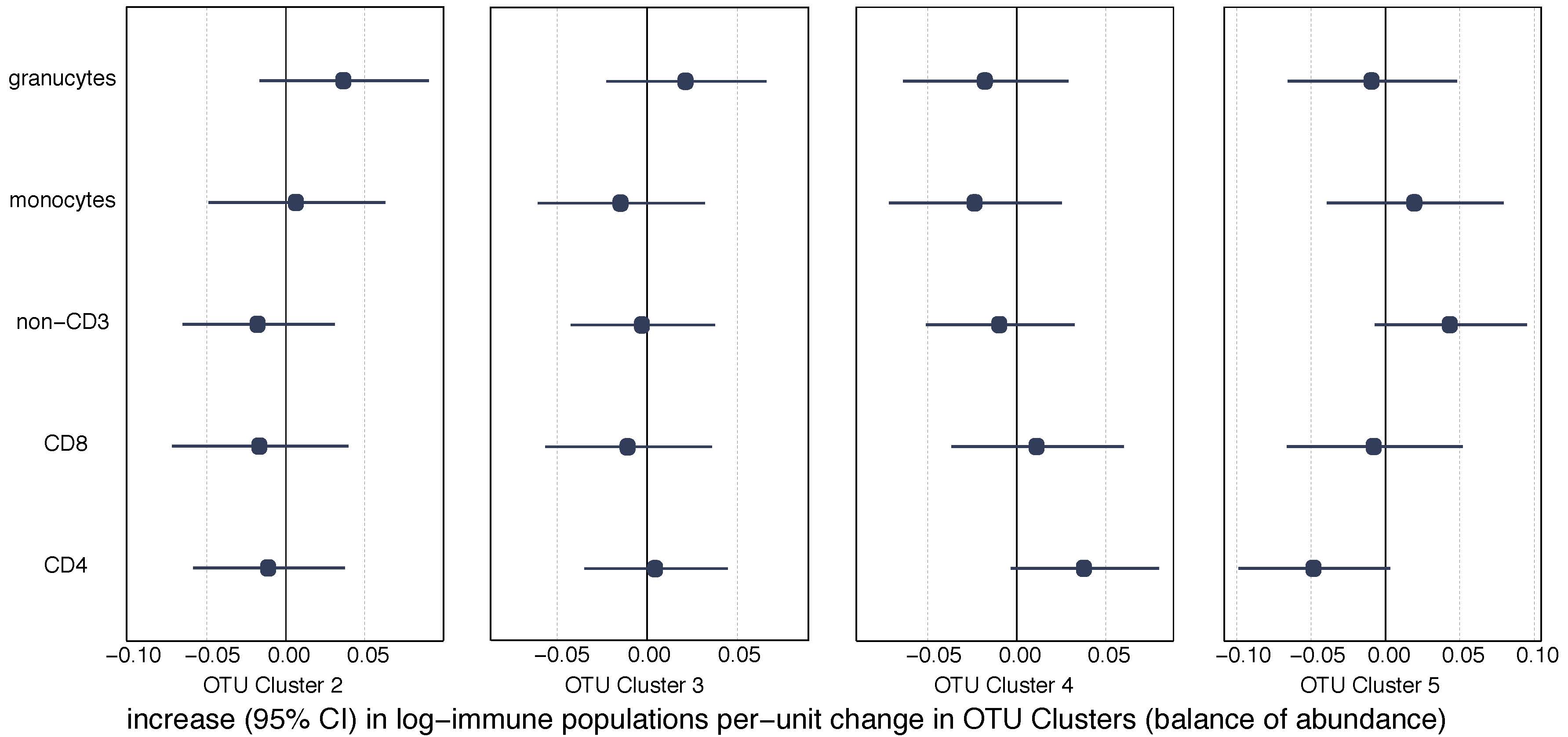

Supplement: Supplementary file 3 [file DataSheet_3.zip › Supplementary Material Presentation/supp. figure 3.jpg]

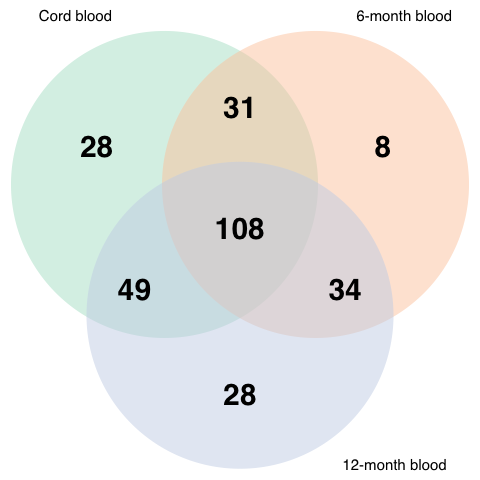

Supplement: Supplementary file 3 [file DataSheet_3.zip › Supplementary Material Presentation/supp. figure 1.jpg]

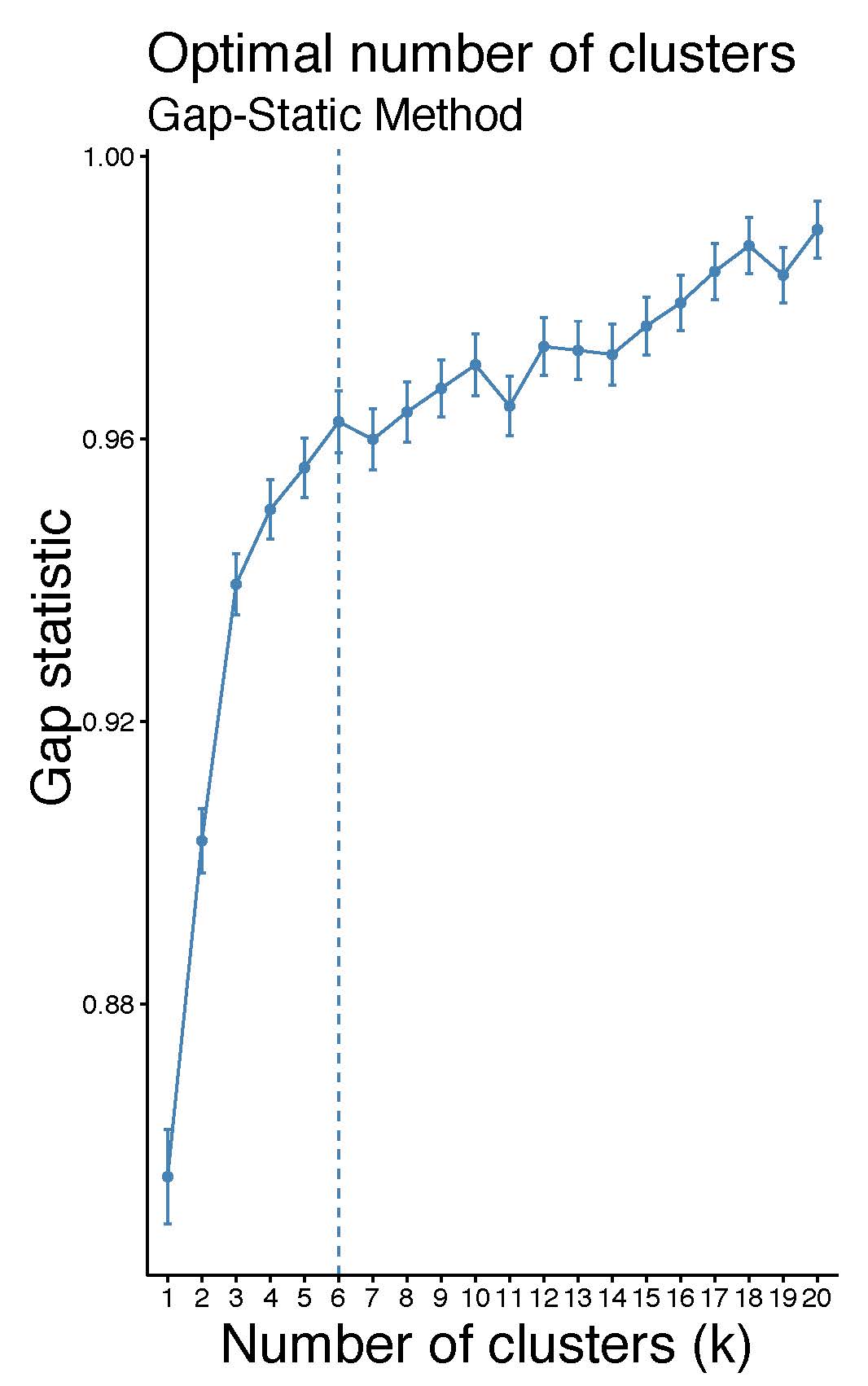

Supplement: Supplementary file 3 [file DataSheet_3.zip › Supplementary Material Presentation/supp. figure 8.jpg]

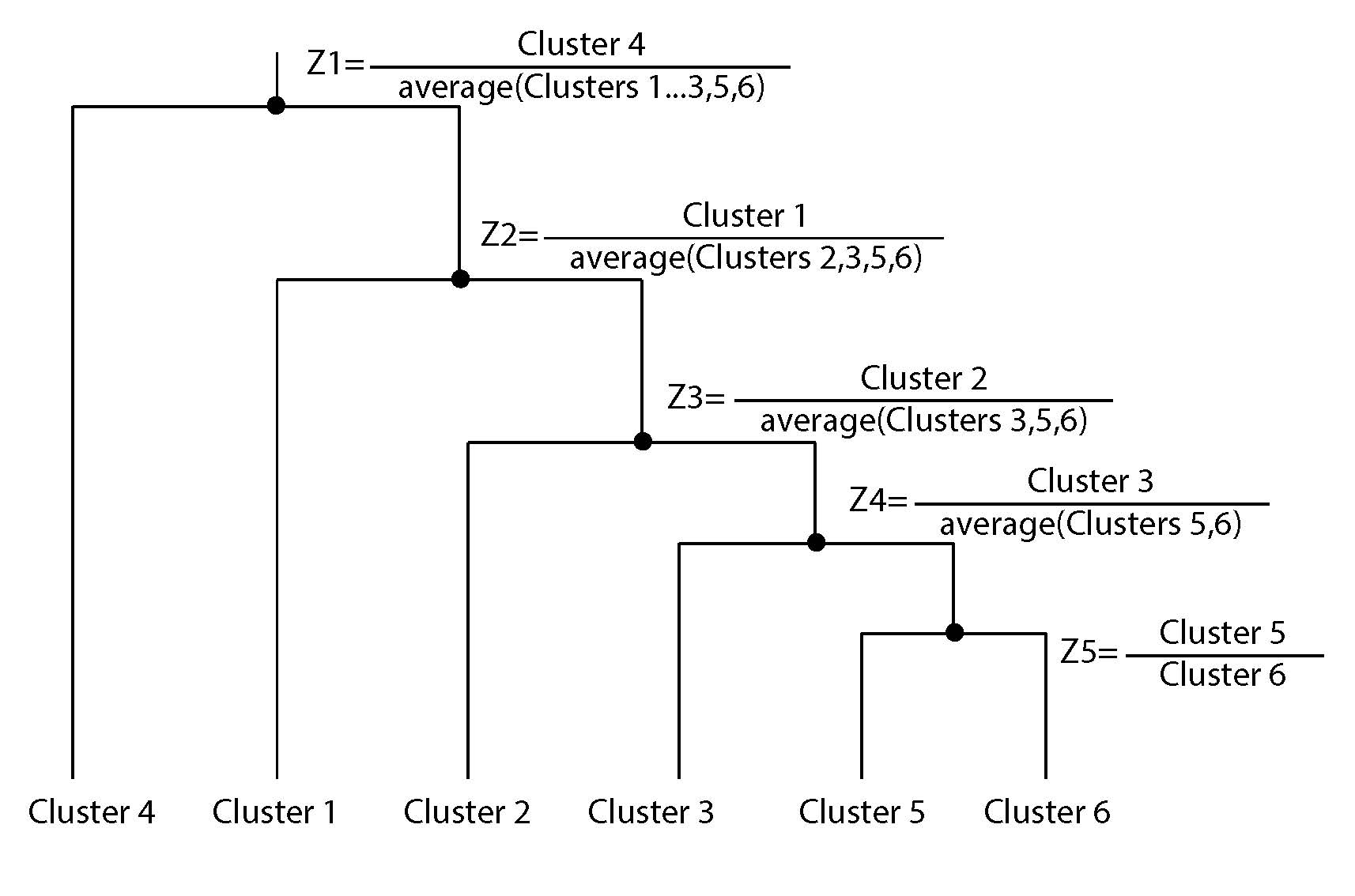

Supplement: Supplementary file 3 [file DataSheet_3.zip › Supplementary Material Presentation/supp. figure 9.jpg]
